# Cyclophosphamide-Induced Morphological Changes in Dental Root Development of ICR Mice

**DOI:** 10.1371/journal.pone.0133256

**Published:** 2015-07-17

**Authors:** Tomomi Kawakami, Yuko Nakamura, Hiroyuki Karibe

**Affiliations:** Department of Pediatric Dentistry, The Nippon Dental University School of Life Dentistry at Tokyo, Tokyo, Japan; University of Palermo, ITALY

## Abstract

**Background:**

Survivors of childhood cancer are at risk of late dental development. Cyclophosphamide is one of the most commonly used chemotherapeutic agents against cancer in children. The aim of this study was to investigate the effects of cyclophosphamide on root formation in the molars of growing mice and to assess the morphological changes in these roots using three-dimensional structural images.

**Methods:**

We treated 16 12-day-old ICR mice with cyclophosphamide (100 mg/kg, i.p.) and 16 control mice with saline. At 16, 20, 24, and 27 days of age, the mandibular left first molars were scanned using soft micro-computed tomography. After scanning, the structural indices were calculated using a three-dimensional image analysis system, and the images were subjected to three-dimensional reconstruction. The length and apical foramen area of all distal roots were assessed. Histological changes in the apical region were then assessed via hematoxylin and eosin staining.

**Results:**

The mandibular molars of all experimental mice showed evidence of cytotoxic injury, which appeared in the form of anomalous root shapes. Although all roots developed further after cyclophosphamide injection, the three-dimensional structural images showed that the roots in the experimental group tended to develop more slowly and were shorter than those in the control group. At 27 days of age, the mean root length was shorter in the experimental group than in the control group. Conversely, the apical foramen of the roots in the experimental group tended to close faster than that of roots in the control group. In addition, hematoxylin and eosin staining of the distal roots in the experimental group showed increased dentin thickness in the apical region.

**Conclusion:**

Our results suggest that cyclophosphamide can result in short root lengths and early apical foramen closure, eventually leading to V-shaped or thin roots.

## Introduction

Modern combination treatment modalities have greatly improved the survival of children with malignant disease. Because of notable improvements in therapies and diagnosis in earlier stages, the survival rate for children with cancer is now approximately 80% [1,2]. With the improving cure rate for childhood malignancies, more attention has been focused on the late effects of cancer treatment in long-term survivors and their quality of life [3,4].

The effects of antineoplastic treatment, particularly alkylating drugs, on the oral health of childhood cancer survivors are known and widely documented [5–7]. Cyclophosphamide (CY), an N-mustard derivative, is an alkylating drug that is widely used in the treatment of cancer because of its capacity to interfere with cancer cell division. However, CY results in important secondary effects caused by nonspecific actions on cells with a high mitotic index, which results in damage to both neoplastic and normal cells [8].

The adverse effects of cancer and cancer therapy during childhood on dental health have been reported in terms of mineralization disturbances, dental caries, crown or root alterations, and delayed or arrested tooth development [4,7,9,10]. Root alterations include premature closure of apices [11,12], blunting of roots [13–15], foreshortening of roots [5,12–14,16,17], delayed and arrested tooth development [12,15,18], and V-shaped or thin roots [5,15,17,19,20]. However, it is difficult to attribute these effects to any single agent or treatment modality, because multimodal therapy is used for almost all childhood cancers.

Animal studies have shown that chemotherapeutic agents induce qualitative and quantitative changes in dental tissues. Alterations in the development of rodent molars as a result of systemic CY administration has been histologically observed [21–23]. However, relatively little is known about the effects of CY on the root morphology of mouse molars up to the stage of apex completion. Therefore, the aim of the present study was to investigate the effects of CY on molar root development in young mice and to assess the morphological changes, particularly in the apical region, in these roots using micro-computed tomography (micro-CT).

## Materials and Methods

### Laboratory animals and experimental design

All animal experiments were conducted in compliance with the Guidelines of the Nippon Dental University, School of Life Dentistry, Section of Biological Sciences, Research Center for Odontology, Tokyo. The study protocol was approved by the Committee of Ethics on Animal Experiments at The Nippon Dental University, School of Life Dentistry, Tokyo.

Thirty-two 12-day-old ICR mice (Clea Japan, Tokyo, Japan) were acclimatized for a week and randomly divided into two groups. The mice were housed with their mothers and provided free access to breast milk and standard solid mouse feed and distilled water ad libitum after weaning. All mice were housed under conventional conditions of controlled temperature (24 ± 1°C), humidity (50% ± 10%), and light (12 h light/12 h dark).

At 12 days of age (postnatal; PN12), 16 mice received a single intraperitoneal injection of CY (Endoxan, Shionogi& Co., Ltd., Tokyo, Japan) at a dose of 100 mg/kg body weight (CY group). As a control group, another 16 mice received injections of an equal volume of saline. The general parameters of mice in all groups, such as body weight and behavior, were observed once a day throughout the experimental period.

At 16, 20, 24, and 27 days of age (PN16, PN20, PN24, and PN27), the mice in the CY group were anesthetized using sodium pentobarbital (Nembutal, Dainihonseiyaku, Tokyo, Japan) and transcardially perfused using 4% paraformaldehyde (Wako Pure Chemical Industries, Osaka, Japan). After fixation, the skull was laterally separated along the epicranial midline suture from the parietal bone over the mandible, and both sections of the mandible in each mouse were dissected.

### Micro-CT analysis

For three-dimensional (3D) examination and visualization of the mandibular first molars, the left first molar was subjected to micro-CT (Ele Scan; Nittetsu Elex, Tokyo, Japan). Image slices were acquired for each sample using a tube voltage of 65 kVp, tube current of 70 μA, and slice thickness of 10.6 μm. Computerized 3D reconstruction was performed using 3D analytic software (TRI/3D-BON; Ratoc System Engineering, Tokyo, Japan). The first molars were extracted and spuriously stained upon the slice image, as described previously by Ide [24]. Thereafter, 3D reconstruction of the tooth images was performed.

The anatomical length of the distal root of each molar was measured along the distal margin of the tooth from the cementoenamel junction to the root apex using 3D analytic software. The apical foramina were observed from the apical side, and the inner margin of the apex foramina of the distal roots was assessed as the apex foramen area using ImageJ 1.46r software [25].

### Histological analysis

Subsequently, the right sections of the mandibular specimens were decalcified using 10% EDTA at 4°C to assess the developmental stage of each sample. Finally, they were embedded in paraffin. Serial sagittal sections with a thickness of 4 μm were obtained using a microtome (RM2065, Leica, Wetzlar, Germany) and stained with hematoxylin and eosin (HE).

**Fig 1:**
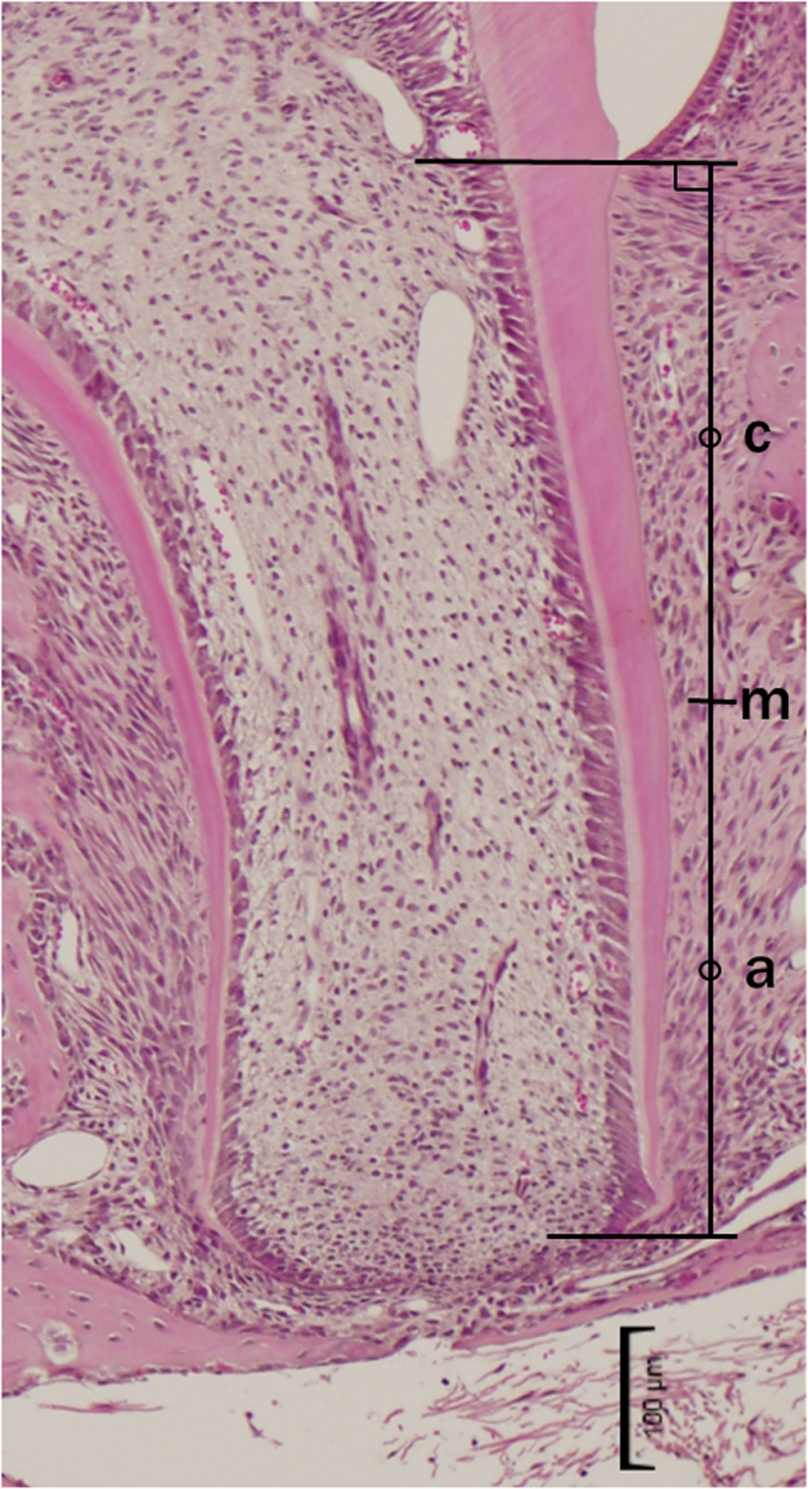
Points used for measuring root length for histological analysis. Hematoxylin and eosin staining shows the distal root of the mandibular first molars at PN16; m, midpoint; c, cervical side; a, apical side. Scale bar = 100 μm. Original magnification, ×4.2.

The anatomical root length of the distal root of each molar on the microscopic images was measured from HE stained sections at PN16 (Fig 1). A straight line connecting the points between the mesial and distal cementoenamel junction was defined as the vertical line. The root length of the distal side of the distal root was the perpendicular line from the vertical line to an apex point, which was the edge of dentine formation. The root length was divided at midpoint "m" into two parts, the cervical side and the apical side of the distal root. The total root length was measured using ImageJ 1.46r software and odontoblasts were counted on the each cervical and apical side of the distal root.

### Statistics

All data are represented as the mean and standard deviation (means ± SDs). Differences between groups were compared using Student’s t-test. Data were analyzed using SPSS statistical software (Statistical Package for the Social Sciences Version 17.0J for Windows, SPSS, Inc., Tokyo, Japan). Student’s t-test was also used to test the association between the two groups. p < 0.05 was considered statistically significant.

## Results

### Shape of the mandibular first molars

**Fig 2:**
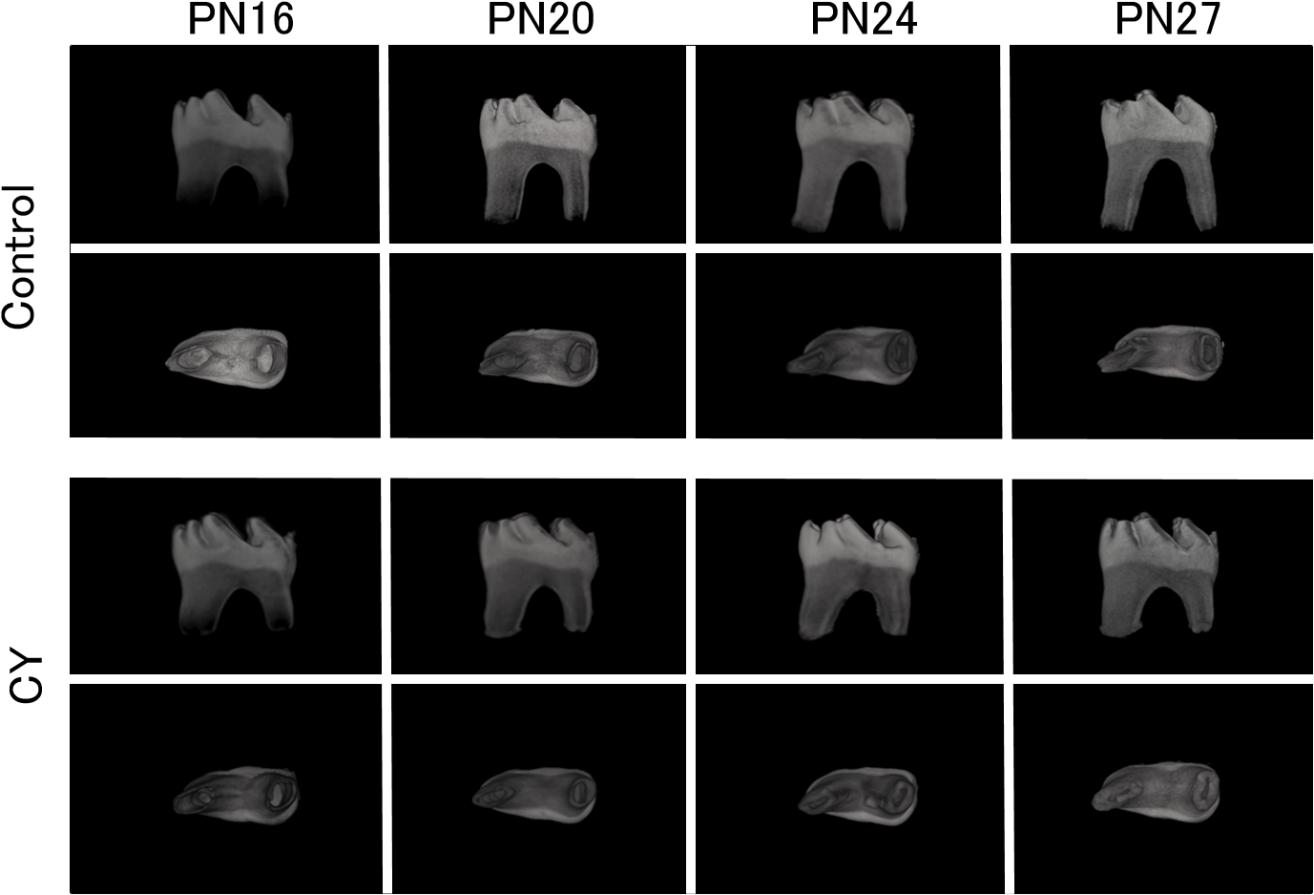
Three-dimensional (3D) structure of the mandibular first molar roots. Micro-computed tomography (micro-CT) images show buccal and apical views of the extracted mandibular left first molars through computerized 3D reconstruction.

Micro-CT was performed to investigate the 3D structure of the first molar roots at PN16, PN20, PN24, and PN27 in both groups. Fig 2 shows buccal and apical views of the 3D structural images generated by computerized reconstruction. Calcification in the furcation area was complete at PN16 in both groups. The shape of the first molars at PN16 was not evidently different between the control and CY groups. In contrast, the 3D images obtained at PN27 for the CY group showed disturbed root elongation compared with the control group. The apical foramina in the control group appeared to become smaller with age on the apical views, although they remained open at PN27. However, the foramina in the CY group showed a more rapid decrease in size compared with those in the control group, with irregular shapes observed at PN27.

### Lengths of the distal roots

**Fig 3:**
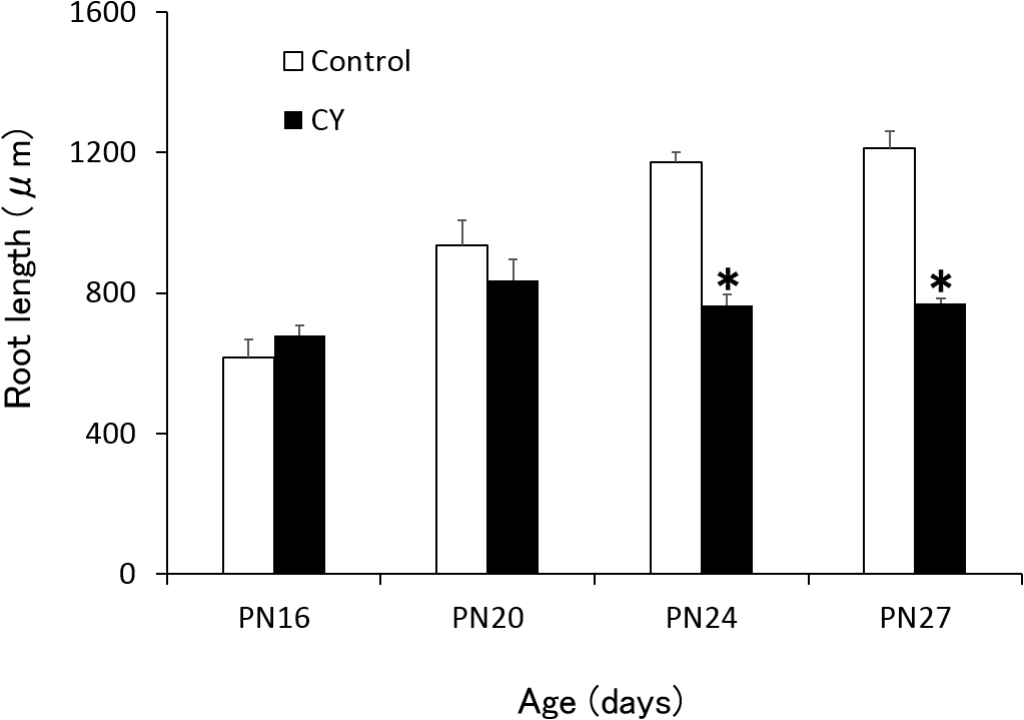
Lengths of the distal roots of the mandibular left first molars. The results are expressed as the mean ± standard deviation (SD). *Statistically significant difference compared with the control (t-test; p < 0.05).

To compare the length of roots between the control and CY groups, the lengths of the distal first molar roots were measured from the cementoenamel junction to the root apex in both groups (Fig 3). The results revealed an increase in root length from PN16 to PN27 in the control group, whereas the length in the CY group showed a slight increase up to PN20 and remained unchanged thereafter. The mean root length at PN16 did not differ between the control (615 ± 52 μm) and CY groups (679 ± 29 μm), whereas that at PN24 and PN27 was significantly shorter in the CY group than in the control group. The mean root length in the CY group (769 ± 14 μm) was approximately 60% of that in the control group (1213 ± 48 μm) at PN27 (p < 0.05, t-test).

### Apical foramen area

**Fig 4:**
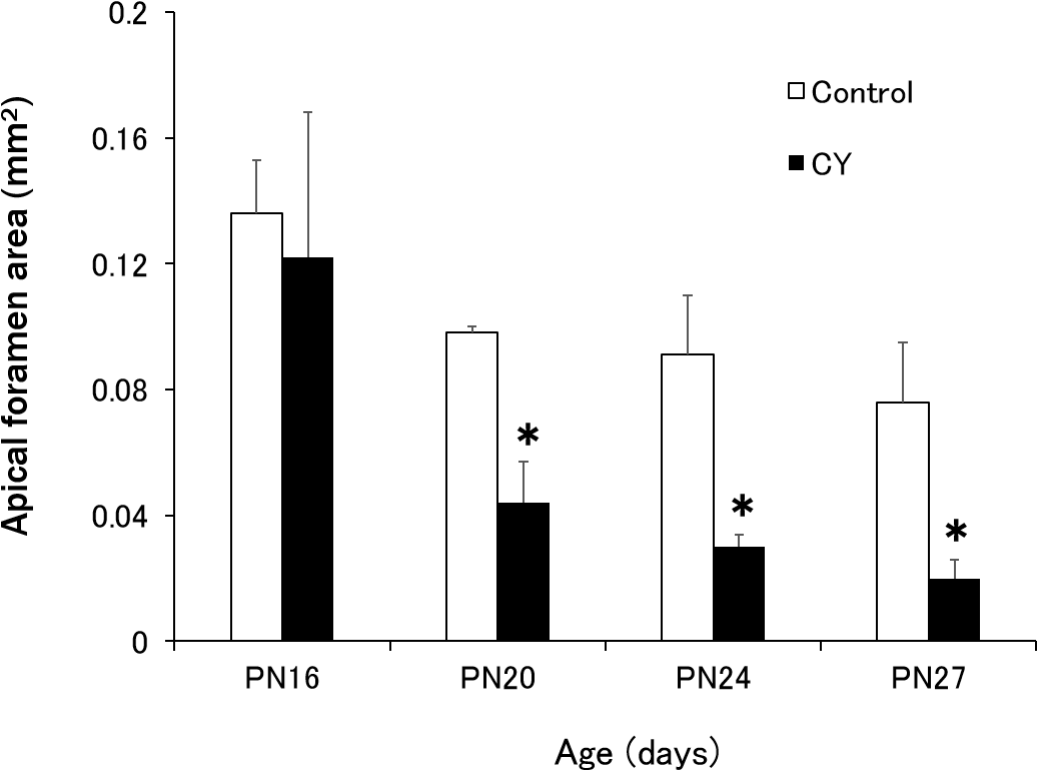
Apical foramen areas in the distal roots of the mandibular first molars. The results are expressed as the mean ± standard deviation (SDs). *Statistically significant difference compared with the control (t-test; p < 0.05).

Fig 4 shows chronological changes in the mean apical foramen area in the distal roots of the mandibular left first molars. The apical foramina in both groups showed a decrease in size from PN16 to PN27. However, a comparison of the area from PN20 to PN27 revealed that the mean apical foramen area was smaller in the CY group than in the control group, with significant differences between the two groups at these time points (p < 0.05, t-test). From PN16 to PN27, the apical foramens in the CY group closed faster than those in the control group.

### Histological analysis

**Fig 5:**
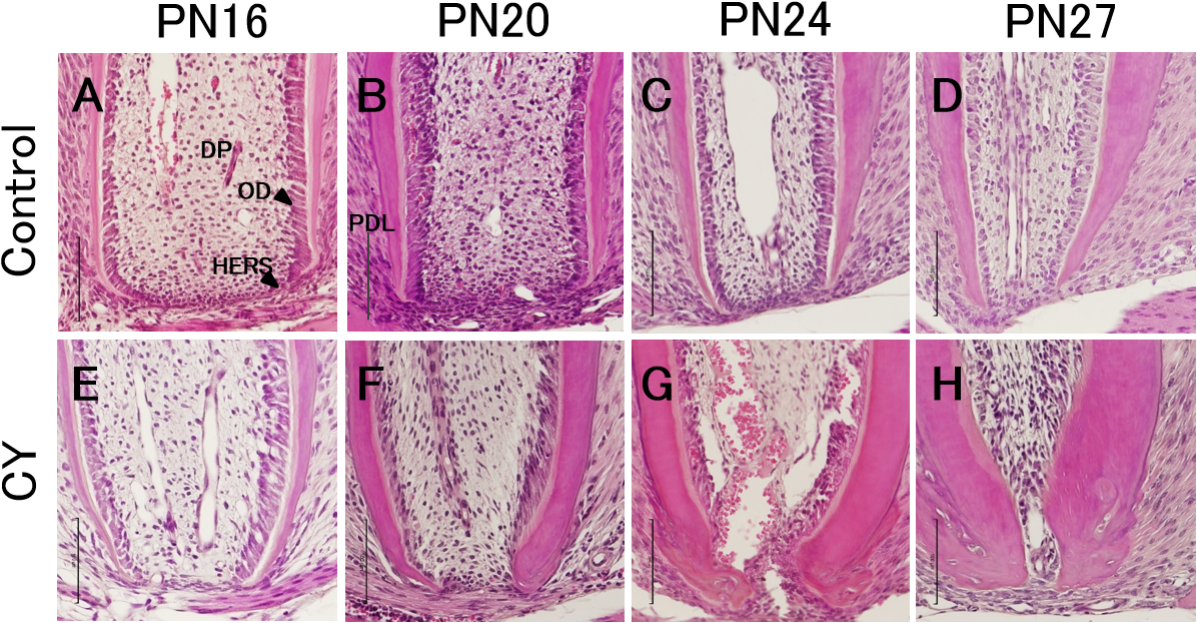
Development of the apical region in the distal roots of the mandibular first molars. Hematoxylin and eosin staining shows the distal root of the mandibular first molars in the control (A–D) and CY groups (E–H). HERS, Hertwig’s epithelial root sheath; DP, dental pulp; OD, odontoblasts; PDL, periodontal ligament. Scale bar = 100 μm. Original magnification, ×20.

To observe the stage of root formation, we examined HE-stained sections of the developing root of the mandibular first molars from PN16 to PN27. Fig 5 shows the histological changes over time in the apical region of the distal root. In both the control and CY groups, the apices of the distal roots were open at PN16, although the cytoplasm of the odontoblasts in the CY group showed initiation of atrophy. At PN16, a cervical loop structure was present at the apex in the control group but not in the CY group. In addition, dentin in the control group formed straight for the distal root axis and connected the cervical loop structure at PN16. In contrast, the odontoblasts in the CY group formed dentin while curving inward toward the central axis of the distal roots. The differentiating odontoblast layer was damaged, and typical columnar odontoblasts were no longer observed in the apical region at PN24 and PN27. Furthermore, HE staining of the distal roots in the CY group showed an increase in dentin thickness in the apical region from PN20 to PN27, whereas the apical foramina were smaller than those in the control group.

Next, to observe the state of odontoblasts and root formation of the roots, we measured the root length and odontoblast number of the distal side of the distal root in HE-stained sections at PN16. Table 1 shows the mean root length and the mean odontoblast number on the distal side of distal root at PN16. The mean root length in the CY group was significantly shorter than that of the control group, and the mean odontoblast number of total distal root in the CY group was lower than that in the control group at PN16. The distal root was divided into two parts, and the mean odontoblast numbers of the cervical and apical sides of each root were compared between the groups. The mean odontoblast number of the cervical side in the CY group showed a slight decrease; however, there were no significant differences between the two groups. In contrast, the mean odontoblast number of the apical side decreased significantly in the CY group.

**Table 1:** Lengths of the distal roots and odontoblast numbers at PN16. p < 0.05, t-test.

	Control		CY		
	Mean	SD	Mean	SD	p-value
Root length (μm)					
whole	793	53	735	26	0.031
Odontoblast number (cells/100μm)					
whole	11.9	1.0	9.9	1.1	0.004
cervical side	11.0	1.8	9.1	1.5	0.062
apical side	13.0	0.8	10.6	1.3	<0.001

## Discussion

On the basis of long-term follow-up studies, survivors who received chemotherapy treatment in childhood carry an increased risk of dental developmental disturbances such as agenesis, root malformation, and microdontia. With regard to morphology, arrested root development resulting in short V-shaped roots and early apical closure are relatively common findings in survivors of childhood cancer [9,15,20]. We analyzed morphological changes in the molar roots of mice using 3D structural images to investigate the mechanism underlying the adverse effects of CY on dental root formation. Our results showed that CY administration in the early stages of root formation resulted in impaired root development and early closure of the apical foramen.

Several clinical studies have provided strong evidence supporting the association between chemotherapy in childhood and dental developmental abnormalities, including tooth agenesis, root stunting, and microdontia [4,26,27]. It is difficult to attribute these effects to any single chemotherapeutic agent because a multidrug regimen is employed for almost all childhood cancers. However, some other clinical studies also suggested that the risk of dental disturbances is higher in patients who receive chemotherapy at less than 5 years of age and in those who receive higher doses of alkylating agents, particularly CY [5,6,28,29].

CY is an alkylating agent known for its capacity to interfere with cancer cell division. However, the toxicity of this drug has been ascribed to a similar effect on normal proliferating tissues. In addition, CY is a non-cell-cycle-specific, dose-dependent, cytotoxic chemotherapeutic agent. Although the 100 mg/kg dose used in this study is higher than that used in low- or standard-risk treatment protocols for cancer in children, it is occasionally used for clinical therapy in certain patient groups, such as high-risk or extremely high-risk groups, depending on the risk or type of cancer [30]. The total dose of an alkylating agent is associated with its cytotoxicity. In clinical protocols, CY is occasionally administered daily. If the total dose of CY exceeds the amount compatible with normal root formation, root malformation may occur. Hsieh reported a dose-dependent association between the cumulative CY dose [6] and Holtta’s Defect Index scores, which include hypodontia, microdontia, and root/crown ratios measured using panoramic radiographs [31].

In a rat model, Näsman revealed that CY administration resulted in decreased root length in the first and second molars and decreased size of the third molars, using histological examination and scanning electron microscopy [23,32]. These findings indicate that the effects of CY are related to the developmental stage of the tooth [23,32]. Kawakami also demonstrated that CY inhibited root growth in the molars of growing mice in a dose-dependent manner; the short roots were not remodeled even after stable occlusion was established [21]. In this study, we performed micro-CT of the mandibular first molar in mice to analyze the morphological changes induced by CY administration. Moreover, to observe the 3D morphology of the dental roots, we used a computer-processing method to highlight only the tooth inside the jaw bone. To the best of our knowledge, this is the first study to show chronological 3D structural changes in dental roots secondary to CY administration in mice. The advantage of using 3D images is that it becomes possible to examine the tooth from all directions. Inhibition of root elongation and acceleration of apical closure in the CY group were apparent on the 3D structural images generated in our study.

The decrease in root length can be explained by the general detrimental effects of CY administration on the mitotic activity in the root proliferation zone [Hertwig’s epithelial root sheath (HERS) or odontoblasts]. It is generally accepted that HERS is important for root formation, and it is also believed that it initiates odontoblast differentiation from the mesenchymal cells in the dental papilla and determines the root shape and size [33,34]. Root development begins under the control of HERS after tooth crown formation through epithelial–mesenchymal interactions. As reported previously, a dose-dependent decrease in root length was observed to result from early loss of HERS [21]. Additionally, Mitomi demonstrated that busulfan, another alkylating agent, resulted in abnormal root development in juvenile rats depending on the stage of administration [22]. It was thus concluded that this abnormal root development resulted from HERS destruction.

In the histological analysis conducted in our study, the normal molar roots had almost achieved their final length without the closure of their apices at PN27, and the root dentin was formed by well-differentiated odontobasts. In contrast, the root length in the CY group was significantly shorter than that in the control group, and it did not reach more than approximately two-thirds of the normal length at PN27 in any specimen (Fig 5). At PN16, the root length in the CY group was already significantly decreased as determined using HE-stained sections, and the odontoblast numbers on the apical side of the distal root had decreased. These results suggest that CY affected the role of HERS and odontoblasts and caused short root malformation. Furthermore, with an increase in the dentin thickness in the apical region, the apical foramen area showed a rapid decrease from PN16 to PN27 despite the inhibition of root growth. At PN16, dentin in the CY group was observed to be formed while curving toward the central axis of the distal roots. Early apical closure occurred because the odontoblast numbers decreased and dentin was formed while curving inward toward the central axis of the distal roots without being regulated by the HERS that was damaged by CY. Anton showed that nondividing columnar odontoblasts and ameloblasts in the rat incisor were not affected by 300 mg/kg CY administered as a single injection [35]. The extent of these abnormalities depends on factors such as the type of cells in the susceptible phases of the cell cycle. Undifferentiated mesenchymal cells are less affected, and differentiated odontoblasts probably have the ability to produce dental tissues even during chemotherapy. The continuous dentin formation of the apical area in the CY group could have been a result of odontogenesis initiated by less-affected odontoblasts. Therefore, V-shaped roots were probably created by this continuous odontogenesis that resulted in apical closure despite the inhibition of root elongation. These results were in accordance with those of clinical reports describing decreased root length and early closure of the apical foramen associated with childhood chemotherapy [6,9,15,26,29].

Various morphological representations such as V-shaped roots, stunted roots, or arrested growth have been reported in previous studies [5,11–20]. These different representations may be a result of variations in the treatment protocols, which included multidrug chemotherapy. Thus, the shape of the root apex may be determined by the rate of root elongation and apical closure, and this rate, in turn, depends on the type and amount of antineoplastic drugs used. Moreover, some factors such as patient age at treatment, type of treatment, and dose of radiation therapy will affect the extent of damage to tooth formation. Remmers observed that the influence of chemotherapy was not the same in all affected teeth in a particular patient. Furthermore, although the size and form of teeth were affected in some patients, only size was affected, with no change in form, in others [36]. The mechanisms underlying dental developmental disturbances are not fully understood because throughout normal dentinogenesis, several signaling molecules mediate the epithelial–mesenchymal interactions required for tooth development. All changes in the surroundings of the developing teeth may interfere with this delicate regulation, leading to different clinical consequences.

In conclusion, the results of the present study may explain some of the tooth abnormalities observed after chemotherapy in humans, although dental development cannot be compared directly between humans and mice. These results show that CY as an antineoplastic drug damages the tooth buds in the early stages of development and is associated with developmental abnormalities in dental roots, including short and V-shaped roots. Although it is difficult to predict the independent effects of each drug used during multidrug chemotherapy on dental outcomes, the impaired root development is irreversible and leads to decreased alveolar bone growth, which results in inhibited vertical development of the lower third of the face [37]. Subsequently, these abnormalities influence function and esthetics in survivors. Education of parents and health care providers about the late dental effects of chemotherapeutic drugs, particularly alkylating agents such as CY, is vital for minimizing the deleterious effects of cancer treatment. Furthermore, survivors at risk of nonpreventable late effects such as microdontia or tooth root stunting should undergo regular follow-ups with a dentist experienced in survivor care.
